# Effect of Treating Depressive Disorders on Mortality of Cancer Patients

**DOI:** 10.7759/cureus.1740

**Published:** 2017-10-03

**Authors:** Cara Sherrill, Melissa Smith, Courtney Mascoe, Elizabeth Bigus, Danielle Abbitt

**Affiliations:** 1 College of Medicine, University of Central Florida

**Keywords:** cancer, depression, oncology

## Abstract

Depression can have debilitating effects on patients with a chronic morbid disease, in particular, cancer. It has been found that patients with a depressive disorder have a poorer prognosis and increased mortality. There is a debate as to whether the treatment of the depressive disorder is beneficial to these patients. Studies demonstrate varying results with pharmacotherapy and behavioral therapy. All cancer patients should be periodically assessed for depressive disorder and the symptoms not dismissed as part of their cancer presentation due to the increased mortality.

## Introduction and background

A depressive disorder, characterized by depression, has a prevalence of 7.6% in the general population over age 12 [1]. Higher rates are reported in populations with additional chronic diseases [2]. A population of particular interest is cancer patients. Cancer is a leading cause of death, with an estimated incidence of 1,685,210 people in the United States in 2016 [3]. A depressive disorder in cancer patients has a reported poorer prognosis [4]. This poorer prognosis is multifactorial with a delayed diagnosis of the depressive disorder [5]. The symptoms of fatigue, sleep disturbance, and decreased appetite are part of the criteria for the diagnosis of depressive disorders; however, these symptoms are often overlooked, as these are commonly expected in cancer patients and may be either dismissed by the patient or overlooked by providers as part of the sequelae of the patient’s neoplastic disease.

Several cancers, including pancreatic, breast, and lung, have been linked to high rates of depressive disorder compared to colon and gynecological cancer. This difference between the types of neoplasm leads to difficulty quantifying the effect of depression on a general population with a cancer diagnosis. Due to the complications with the diagnosis of depression in cancer patients, there is little reliable evidence as to the percentage of patients affected. Studies have reported 0-38% of cancer patients have a concomitant diagnosis of a major depressive disorder with 0-58% of cancer patients at least on the depression spectrum [6].

Several trials and studies have been conducted to investigate the effect of the treatment of depressive disorders on cancer patient mortality. Studies looking at cancer populations with a previously reported higher prevalence of depressive disorder were preferentially selected in order to focus on those most likely at risk. These studies have been grouped by type of cancer: lung, breast, head and neck, and glial and colorectal. Additionally, a meta-analysis of unspecified cancer is included.

## Review

The Pubmed and Cochrane libraries were searched within the time period of 2009 to 2017.

A 2012 clinical trial by Pirl et al. evaluated depressive disorders and survival in metastatic non-small-cell lung cancer (NSCLC) patients with early palliative care (EPC). A total of 151 patients with newly diagnosed (≤ eight weeks of diagnosis) NSCLC were randomized into a treatment group with EPC and a control group that received standard oncology care alone. EPC included meeting with the palliative care team within three weeks of diagnosis and at least monthly until death. Patients in the standard care group could access palliative care services on request. Patient Health Questionnaire-9 (PHQ-9) scores were assessed at baseline and, after 12 weeks to diagnosis, presumed major depression syndrome (MDS). Three sensitivity analysis models were used to account for patients who died before 12 weeks. This trial demonstrated a shorter median survival time for patients with MDS at baseline (five months) compared to no MDS at baseline (10 months). MDS was associated with worse survival (HR, 1.82; 95% CI, 1.10-3.01). Patients with MDS who received EPC had a greater depression response at 12 weeks than patients with MDS who received standard oncology care (42.9% v 0%; *p* = .04 ) [7]. No patients in the standard group had a response at 12 weeks. While this study reports a significant worsening in survival in patients with MDS, only one statistical model demonstrated this significant association between changes in depression and survival. This model was used on patients who died before 12 weeks and calculated a projected change in PHQ-9 at 12 weeks. This model differed from the other models used in that it also reported that baseline depression is associated with worse survival, which does not support the use of EPC. A potential explanation for this finding is that a disease in depressed patients may be more aggressive and/or treatment-resistant. This study has several limitations, including its modest sample size, lack of diagnostic interviews of presumed depressed patients, infrequent measures of depression (baseline and 12 weeks), and failure to evaluate coping styles. Regardless of these limitations and data inconsistency, this study demonstrates that comorbid disease, such as depressive disorder, is treatable in NSCLC and should be treated due to the poor quality of life it can cause for cancer patients. EPC offers additional treatment options for providers.

A 2013 article by Sullivan et al. evaluated the effect of depression treatment on the mortality of veterans with lung cancer. This study included 3,869 patients between the years 1995 and 2010. Fourteen percent were diagnosed with a depressive disorder within two years prior to lung cancer diagnosis. Survival was compared between lung cancer patients with and without a diagnosis of depressive disorder. Cancer type: non-small cell lung cancer (NSCLC) and small cell lung cancer (SCLC), and stage (I-IV) were identified and stratified across both groups. Variables such as radiation therapy and chemotherapy, surgery, tobacco use, and distance to the nearest medical center were also considered as confounding factors and were studied. Several treatment modalities were utilized, including antidepressant medications, hospitalizations, and mental health clinic visits. A diagnosis of depressive disorder was linked to poorer survival outcomes, regardless of the stage of disease (HR = 1.14, 95% CI: 1.03-1.27) [8]. Diagnosis of depression was not significantly associated with radiation therapy, surgery, or chemotherapy. The effect of depression treatment on mortality may be due to the increased severity of depression correlating to the greater use of treatment for depression. As is consistent with existing literature, here it is found that depressive disorder is a poor prognostic factor in the cancer population.

In a later 2016 article, Sullivan et al. revisited the question of depression and survival of lung cancer patients in a national prospective, observational cohort study. Participants meeting the selection criteria who completed both a baseline and follow-up survey at 12 months were included. The cohort was composed of 1,790 participants, 681 (38%) of which had depressive disorders at baseline. The primary outcome of the study was defined as patient survival. Participants were separated into four groups: (1) never depressed, (2) remission of symptoms, (3) new onset depressive disorder, and (4) persistent symptoms. Baseline depression symptoms were associated with increased mortality rates in participants with early-stage disease (stage I-II) (HR = 1.61, 95% CI: 1.26-2.04), but not late-stage disease (stage III-IV) (HR = 1.05, 95% CI: 0.91-1.22). A follow-up survey demonstrated that depressive disorder was associated with increased mortality in both early and late-stage disease (HR = 1.71, 95% CI: 1.27-2.31 and HR = 1.32, 95% CI: 1.04-1.69). Remission of depression symptoms at follow-up proved to be associated with comparable mortality, as never having had any depressive symptoms (no increased risk of mortality). The never-depressed and remission groups had a median survival that was 130 days greater than those in the new onset depressive disorder and persistently depressed groups [9]. The findings of this study suggest the potential reversibility of the effects of depression symptoms on the outcome of cancer patients.

Valachis et al. explored the relationship between selective serotonin reuptake inhibitor (SSRI) use and adherence to endocrine therapy and breast cancer mortality. This nested case-control study collected data from three breast cancer registries in Sweden. The study followed 18,432 women diagnosed with estrogen receptor (ER) positive breast cancer, with non-distant metastases from July 2007-2011, on tamoxifen or aromatase inhibitors. A total of 445 (2.4%) patients died of breast cancer as the cause of death by July 2011. Patients were categorized further regarding oral endocrine therapy adherence of over 80%, calculated based on the medication possession ratio (MPR). Patients with MPR over >80% were categorized as adherent and those with MRP <80% as patients with low adherence. The authors found that women on SSRIs had lower adherence to endocrine therapy whether having initiated SSRI prior to breast cancer diagnosis (OR 1.33; 95% CI 1.03-1.73) or starting for the first time after diagnosis (OR 1.37; 95% CI 1.01 – 1.85). Patients who had endocrine therapy and SSRI use overlapping <50% of the study period had a higher risk of low adherence. However, this association was no longer apparent when patients overlapped therapies >50% of the study period. Next, the association between short-term SSRI use at the same time as tamoxifen therapy regardless of adherence to oral endocrine therapy was explored. It was found that patients with concomitant short-term SSRI use actually had worse breast cancer survival than those who did not use SSRI (adjusted OR 1.58; 95% CI 1.00-2.49). However, no difference in mortality was found when no SSRI use was compared with long-term SSRI use (adjusted OR 0.68; 95% CI 0.29-1.57). Interestingly, when the study analysis was restricted to only women with >80% adherence to therapy, neither short-term (OR 1.58; 95% CI 0.74-2.68) nor long-term (OR 0.85; 95% CI 0.35-2.08) SSRI use differed from no SSRI use on breast cancer mortality [10]. Overall, this data suggests that women who take SSRI initiated either before diagnosis or after have lower adherence to endocrine therapy when the SSRI prescription period overlaps with the therapy <50% of the time. However, those women who were prescribed SSRIs for longer periods of time had no difference in their endocrine therapy adherence rate. It is possible that women who are on antidepressants for shorter periods of time are not adequately treating their depressive disorder and, thus, are less likely to adhere to breast cancer treatment as depressive disorders have been shown to reduce adherence rates. It appears that while women with depressive disorder and breast cancer are less likely to adhere to their adjuvant therapy, those adequately treated for longer periods of time are no different than breast cancer patients without a depressive disorder in terms of mortality. The authors point out that SSRI use in more than 50% of the study period corresponded with more than one year of SSRI use. If this is indeed the case, it is important to emphasize to breast cancer patients with depressive disorders the necessity of long-term treatment with SSRI. This study had several limitations. First, adherence was defined using prescription refills, which does not equate to patients actually consuming the medication. It also does not account for patients that may have picked up their prescription, started to use it, and then decided to stop taking it. Thus, the non-adherence group could have actually been underestimated. Second, SSRIs can be used for conditions other than depressive disorders such as anxiety disorder, hot flashes, or eating disorders. Third, this study was limited in that it only analyzed the use of SSRIs and did not examine the use of other antidepressants or non-pharmacological depression treatments.

A study published in 2010 in the American Society of Clinical Oncology explored the relationship between depression symptoms and survival time in patients with metastatic breast cancer. This secondary analysis of a randomized trial looked at the effect of supportive-expressive group therapy on depression symptom scales compared to the control group who solely received educational materials on depression. The study included 125 women with documented metastatic or recurrent breast cancer. All women were given educational materials and completed the Center for Epidemiologic Studies-Depression Scale (CES-D) at the start of the study and then again at four, eight, and 12 months. This scale included 20 common affective and somatic symptoms of depressive disorders and had women rate them on a scale from zero (rarely) to three (most or all of the time). A total of 64 women in the intervention group received one year of supportive-expressive group therapy (SET). The researchers then analyzed the effect of SET and depression symptoms on survival for up to 14 years. A decrease in CES-D score over one year was significantly associated with longer survival over a 14-year follow-up period (n=101; HR, 1.68; 95% CI: 1.16-2.45). There was no significant effect of treatment condition. When the investigators removed the last CES-D scores from the analysis before death in order to control for the effects of pre-terminal depression or the effects of early death in a certain group, there was still a significantly longer survival time (n= 93; HR, 1.54; 95% CI: 1.05-2.26) [11]. This data suggests that decreasing depression symptoms correlates with an increasing survival time. Given the lack of effect of SET on the outcome, it appears that the means by which patients’ depression symptoms were reduced do not impact survival. The reduction in depression symptoms over only the first year had effects on survival even years later. This emphasizes the importance of managing depression symptoms as soon as possible, as the impacts of reducing depressive symptoms are long-lasting. There are several limiting factors to this study. It is possible that women with better prognoses and, therefore, better survival times have an easier time reducing depression symptoms. There were confounding variables, such as diet and exercise, which have been documented to improve both depression symptoms and long-term survival time. Additionally, those with cancers that become more aggressive over time may have required more chemotherapy or radiation, which could have impacted depression scores.

A recent 2017 article by Rieke et al. utilized the surveillance, epidemiology, and end results (SEER) program to evaluate the impact of depressive disorders on five-year survival rates in individuals diagnosed with head and neck cancers (HNC). Numerous participant characteristics were compared against the presence or absence of diagnosed depressive disorder, further separating individuals diagnosed with depressive disorder prior to or after the cancer diagnosis. HNC, along with their treatments, affect personal appearance, quality of life, and daily functioning, which have been linked to high rates of depressive disorders in this population. When looking at cancer-specific mortality, this study showed that lifespan in individuals without a diagnosed depressive disorder was approximately 28 months longer than individuals diagnosed with a depressive disorder either before or after their cancer diagnosis. Cancer-related deaths were also found to be 41% higher in individuals diagnosed with depressive disorders after their cancer diagnosis than in those without a diagnosed depressive disorder. When analyzing all-cause mortality, those diagnosed with a depressive disorder after their cancer diagnosis had a mortality rate almost 1.4 times those without a depressive disorder (HR = 1.38; 95% CI: 0.4-0.57) [12]. While the results of this study were significant, several limitations have an unknown impact. There is no information on what methods were used to evaluate for depressive disorders and it excludes individuals with either undiagnosed depressive disorders or symptoms that do not meet diagnostic criteria. Information was not provided on depressive disorder treatment received and symptoms were not followed throughout the course of their disease. Data used was from the SEER-Medicare database, which limits the study participant population to age 65 and above. Despite these limitations, identifying the correlation between depressive disorder and decreased survival has helped highlight the need for adequate screening to identify depressive disorders early and provide appropriate treatment and support.

A 2012 article by Walker et al. assessed tricyclic antidepressant (TCA) use on the survival outcomes in patients with either glioma or colorectal cancer; it is the first-known large-scale study evaluating this topic. This study evaluated 1,364 glioma patients and 16,519 colorectal patients from 1987-2010, who received TCA treatment for greater than six months' post-cancer diagnosis. Patients were divided into two groups based on their mean dosage of TCA received. Cancer stage, grade, and patient lifestyle (diet and exercise) were not collected and studied. The timing of TCA exposure: exclusive pre-diagnosis, pre-diagnosis and post-diagnosis, and exclusive post-diagnosis was evaluated. For glioma patients, there was a non-significant decrease in the hazard ratio (HR) for post-diagnosis TCA exposure and no association with mortality outcomes for exclusive pre-diagnosis exposure. For colorectal patients, post-diagnosis TCA exposure was associated with a poorer prognosis (HR = 1.37; 95% CI = 1.21-1.54), and no association was found for exclusive pre-diagnosis exposure [13]. A possible explanation for this finding is the use of low-dose TCA for chronic pain management, which may be linked to more advanced disease and, thus, poorer prognosis. Despite laboratory evidence that TCAs have anticancer properties, this study failed to demonstrate a benefit of the use of TCA in regards to survival outcomes; in fact, the data indicate that the use could be detrimental in colorectal cancer patients. Any future studies should not include colorectal cancer patients; glioma patients may still be considered as there was a small, but insignificant, increase in survival. At this stage, it is not clinically sound advice to offer TCA treatment with the expectation of prolonging survival.

Satin et al. evaluated the extent to which depressive symptoms and major depressive disorders in cancer patients might predict disease progression and mortality. Through a literature review, the authors used prospective studies to assess the association between depressive symptoms and the risk of disease progression and/or mortality in cancer patient populations. Inclusion criteria for the required specific examination for depressive symptoms or the diagnosis of major/minor depressive episodes or disorders performed after the cancer diagnosis. Articles were excluded if they did not utilize standardized reporting tool, data were insufficient, depression was assessed before cancer diagnosis, or if the study measured the effect of an intervention. A reference list of review articles was considered to ensure relevant studies were included. Ultimately, 33 studies were included. The combined unadjusted relative risk (RR) calculated from the included studies regarding the effect of depressive symptoms on mortality in cancer patients is 1.25 (95% CI: 1.12-1.40). The combined HR was 1.05 (95% CI: 1.01-1.09). When the data were analyzed by the length of follow-up, it is shown that studies that assessed mortality after less than five years demonstrate significant results (RR = 1.35; 95% CI: 1.13 – 1-61), (HR = 1.05; 95% CI: 1.01-1.09). Studies that assessed mortality after five years or later similarly yielded significant results. An assessment of the effect of clinical depression on mortality in cancer patients provides mixed results, with an overall RR (unadjusted), including three studies is 1.39 (95% CI: 1.03-1.89) [14]. The meta-analysis performed suggests that depression status in cancer patients may serve as a modest predictor of mortality, but the variability of the studies included make it difficult to reach a firmer, more generalizable conclusion.

## Conclusions

Depressive disorders can have a profound impact on quality of life, which, in turn, affects an individual’s overall morbidity and mortality. It is without surprise that a depressive disorder in conjunction with a chronic or terminal illness, such as cancer, will have continual effects on both quality and quantity of life. These observations have led to a wide study of the effects of depressive disorders along with the effects of treatment on morbidity and mortality after a cancer diagnosis. In this paper, numerous studies looked at the effects of depressive disorders and their treatment on various types of cancer.

In individuals with lung, head and neck, and breast cancer, the diagnosis of a depressive disorder seemed to correlate with poorer outcomes and increased mortality. As shown in these studies, considerable variation was experienced, depending on the type/location of cancer, the stage and extent of the disease, co-morbidities, and several other patient factors. Therefore, it is difficult to draw conclusions that would benefit all cancer patients as a whole, and future studies to look at specific cancers is recommended.

Clinical evidence demonstrates that the effects of depressive disorders can be long-lasting, which highlights the importance of managing depression symptoms as soon as possible. As far as which depressive disorder treatment to choose, the impact of different treatments on mortality in cancer patients needs to be further explored. Just as when treating depressive disorders alone, many patient factors play a role in developing a personalized and appropriate treatment plan. For instance, in colorectal cancer, one study found the treatment of depressive disorders with tricyclic antidepressants post-cancer diagnosis not only failed to improve overall mortality but seemed to increase mortality.

Despite the limitations found within these studies, a correlation between depressive disorder and survival has been identified, and for most types of cancer, that correlation is with decreased survival. This link between depressive disorder and decreased survival has highlighted the need for adequate depressive disorder screening both early and throughout care to provide appropriate treatment and support when needed.
